# The Role of Bioacoustic Signals in Koala Sexual Selection: Insights from Seasonal Patterns of Associations Revealed with GPS-Proximity Units

**DOI:** 10.1371/journal.pone.0130657

**Published:** 2015-07-08

**Authors:** William Ellis, Sean FitzGibbon, Geoff Pye, Bill Whipple, Ben Barth, Stephen Johnston, Jenny Seddon, Alistair Melzer, Damien Higgins, Fred Bercovitch

**Affiliations:** 1 School of Agriculture and Food Science, The University of Queensland, Brisbane, Queensland, 4072, Australia; 2 San Diego Zoo Global, P.O. Box 120551, San Diego, California 92112–0551, United States of America; 3 Faculty of Veterinary Science, The University of Queensland, Brisbane, Queensland, 4072, Australia; 4 CQUniversity, Koala Research Centre of Central Queensland, School of Medical and Applied Sciences, Rockhampton, Queensland, 4702, Australia; 5 Faculty of Veterinary Science, The University of Sydney, Sydney, Australia; 6 Kyoto University Primate Research Institute & Wildlife Research Center, 41–2 Kanrin, Aichi, Inuyama, 484–8506 Japan; Universidad Nacional Autónoma de México, MEXICO

## Abstract

Despite being a charismatic and well-known species, the social system of the koala (*Phascolarctos cinereus*—the only extant member of the family *Phascolarctidae*) is poorly known and much of the koala’s sociality and mating behaviors remain un-quantified. We evaluated these using proximity logging-GPS enabled tracking collars on wild koalas and discuss their implications for the mating system of this species. The frequency and duration of male-female encounters increased during the breeding season, with male-male encounters quite uncommon, suggesting little direct mating competition. By comparison, female-female interactions were very common across both seasons. Body mass of males was not correlated with their interactions with females during the breeding season, although male size is associated with a variety of acoustic parameters indicating individuality. We hypothesise that vocal advertising reduces the likelihood of male-male encounters in the breeding season while increasing the rate of male-female encounters. We suggest that male mating-season bellows function to reduce physical confrontations with other males allowing them to space themselves apart, while, at the same time, attracting females. We conclude that indirect male-male competition, female mate choice, and possibly female competition, mediate sexual selection in koalas.

## Introduction

Sexual selection produces differences in reproductive success arising from competition for mates and/or choice of mates [1,2]. As a result, research has concentrated on assessing the roles of two factors: female mate choice (selecting mates on the basis of attractiveness) and male-male competition (defeating or deterring rivals for mating opportunities). One consequence of both factors is a tendency to produce sexual dimorphism, for example features of one sex such as ornaments that do not appear to have a role in helping the animal survive. Variation in body size and vocal parameters between males and females of the same species are other examples. In koalas (*Phascolarctos cinereus*), males tend to be larger than females, are significantly more vocal and possess sternal scent glands that are absent in females [3][4][5,6].

However, because of the koala’s cryptic and asocial nature, observations of copulations or male-male aggression in this species are only occasionally reported [7,8]. Male bellowing increases early in the breeding season (September—December) and is hypothesized to mediate female mate choice in koalas [4]. Charlton et al. [9] have suggested that male size-dependent vocal cues are advertisements used by females to exercise mate choice. Male koalas have higher variance than females in reproductive output [10], but male body mass only contributes to one-third of the variance in fecundity. Head length is associated with vocal tract length [11], so associations between male head length and bellow characteristics are expected, but the extent to which these characteristics influence male-female encounters in the wild remains unexamined.

Whether male koalas have higher variance in the number of mates than females is unknown, but since females usually produce a maximum of one offspring per year, there is more likely to be a correlation between number of mates and number of offspring for males compared to females. We previously investigated the intensity of sexual selection in koalas, finding Crow’s Index of Selection [12,13] (which places the variance in reproductive output over the calculated mean reproductive output to provide a ratio indicating an index of sexual selection [12,13]) in female koalas to be 1.294, while that of males is 3.852. We also found the coefficient of variation in annual reproductive output among females to be 9.1%, compared to 19.2 in males [10], indicating that opportunities for sexual selection to mediate variance in female reproductive output are less than in males, but not nonexistent. Here, we use our data on social behavior of koalas to test whether males do have greater variability in number of mates than females [14], which could point to the strength of sexual selection [13] in this species.

Little is known about the mating habits of koalas: reports of breeding behaviour are rare and, when available, are commonly of small sample sizes and refer to observations of social interactions [8], are based on captive subjects [15,16] or, are drawn from genetic analyses that inform field observations [17]. Across their range, koalas are seasonal breeders [18,19] and between September and December the sonorous bellow of the males can be heard at night throughout the forests and woodlands that they inhabit. Because they reside in trees, agonistic interactions between koalas are potentially fatal through falls, so the potential exists for vocal encounters to replace physical confrontations since vocalizations carry accurate indicators of size [9]. No data informing this aspect of their sociality have been presented to date.

By using on-animal proximity-GPS units, we sought to examine several aspects of koala sociality and provide information relative to several hitherto untested predictions concerning sexual selection in the koala:

1. If male competition were mediated by agonistic interactions, then we would expect male-male encounter rates to be higher during the breeding than the non-breeding season [20]. Alternatively, if male vocalisations mediate this competition, then we would not expect a significant difference in male-male encounter frequency between seasons.
2. Variability in reproductive success is higher for male than female koalas [10]. Our prediction is therefore that the number of females encountered by males in our sample will vary more than the number of males encountered by females.
3. If vocal advertising for mates results in larger males encountering more females than smaller males (in the case where females select the larger males on the basis of their bellow [21]), we expect to find a relationship between male body size and the number of male-female interactions.
4. If female koalas respond to male calls originating outside of their normal range area [4] and seek males when in oestrus [22], female home ranges would be expected to increase during the breeding season compared to the non-breeding season.
5. We expect, out of necessity for mating, male-female encounter rates (or their duration) to increase in breeding compared with non-breeding season. However, the extent of the koala’s otherwise solitary nature has not previously been examined. Our technology allows us to examine whether both male and female koalas are indeed solitary. The results of this examination will determine whether we can test Sharpe’s [23] prediction of higher mutual avoidance amongst males relative to females. If all koalas universally avoid one another except when mating, we will not be able to examine Sharpe’s prediction.

## Methods

### Study site and koala population

St Bees Island (20° 55′ S; 149° 26′ E) is a continental island lying 30 km north-east of Mackay off the central Queensland coast of Australia. Comprising some 1000 ha, this island is part of the South Cumberland Islands National Park. The island’s vegetation consists of a mosaic of forest, woodland and grassland over steep, volcanic hills [24].

A founder group of between 12–17 koalas [25] translocated from the mainland in the late 1930’s established the island population that is now estimated to number in the hundreds [26]. This population has been the subject of a broad range of ecological investigations, including habitat selection [27], spatial dynamics [28], genetic diversity [29] and sexual selection [10]. The island is relatively undisturbed due to the absence of habitation by humans and the only known predators are birds of prey such as the Wedgetail eagle, *Aquila audax* [30]. Disease has been recorded from some koalas on the island, including the bacterial pathogen *Chlamydia* [31].

### Study animals

Twenty-one koalas in the main 20 ha study area (the knoll; see Ellis et al., [28]) were located by our team of 5–7 experienced koala spotters that systematically searched the knoll during five consecutive days in September 2012. All koalas that were encountered were captured and fitted with radio tracking / proximity logging collars and as the majority of the knoll was searched every day, the proportion of new koalas located on each day reduced over the course of the week. Only two non-collared koalas were located on the fifth day indicating that the site was saturated with collared koalas.

Koala captures were completed with a modification to the method of Ellis *et al*. [32]: we fitted a plastic bag (40 cm x 20 cm, volume 8 l) to the end of a telescopic aluminium pole and waved this above the koala’s head, which resulted in the koala descending the tree, presumably to avoid the noise and movement. Once on the ground, the koala was placed in a cloth bag (40 cm x 80 cm, volume 20 l). While restrained in the bag the koala was anethsetised (isoflurane 5% induction, 2% maintenance as per McGowan et al [33]) and subjected to a veterinary examination. Once anethsetised, each adult koala (>4 kg) was fitted with a proximity / VHF tracking collar (Sirtrack Pty Ltd, New Zealand) containing a uniquely coded UHF transmitter and a UHF receiver and recorder that logged all other UHF frequencies used in this study. The receiver was set to detect and record other UHF frequencies emitted within 1 m of the receiver, meaning that two koalas were required to be within this proximity for an interaction to be recorded. On average, inter-tree distances were 10 meters, so a proximity reading of 1 meter would indicate that the two collar-wearing koalas were in the same tree. When two collared koalas were within the detection distance, both proximity loggers recorded the unique code of the other collar, as well as the start time and duration of the interaction.

The VHF transmitter allowed radio-tracking of collared koalas, and a small GPS data-logger (Mobile Action, Taiwan) was also fitted to the collar, programmed to record geographical position every 10min between 23:00 and 24:00 daily for two months (60 days). Only the recorded locations with an estimated horizontal precision error (EHPE) output of 0–15 m were used in analyses (77% of all fixes), as tests with stationary GPS units revealed that this threshold resulted in an average accuracy of 10.0 ± 0.24 m from the actual point (measured using a backpack-mounted differential mode GPS accurate to less than 1 cm). Only the single most accurate daily fix for each individual was included for analysis. Total collar weight ranged from 140–150 g and included a rubber weak-link preventing injury or death by entanglement of the collar.

In May 2013, the collared koalas were radiotracked and recaptured for examination, data downloading and re-collaring. As in September 2012, transect searches of the knoll were repeated to ensure that all koalas present at the study site were fitted with collars (including individuals that had immigrated since September 2012). All adult koalas that were observed were caught and at the end of the five-day period a total of 18 koalas were collared. In September 2013, all koalas were again caught, their collars were removed and the koalas were released at the point of capture.

### Data analyses

Because our collars accurately recorded all occasions during which one collar detected another (within one meter), we have partitioned our data into “contacts”, defined as the exact time and date when two koalas came within this range (“in contact”), and “encounters” defined as the sum of the time during which collars were recording each other’s presence (i.e. the length of each contact). Repeated contacts not separated by more than 15 min were considered as an individual contact and encounter; when no contact was recorded for at least 15 min, further contacts were considered to be unique encounters. Our time threshold was based on our observations of social interactions in wild koalas at the study site and was used to try and account for occasions when two koalas were associating with each other but they temporarily moved outside of UHF detection distance (1 m) for short periods. Thus, we could distinguish between contact frequency (the number of contacts) and encounter duration (the period of time during which contacts not separated by more than 15 min were recorded) of proximity between koalas.

To assess differences in social interactions during breeding and non-breeding periods of the year, analysis of interaction data was restricted to two three-month periods: mid-Sept to mid-Dec 2012 (breeding season) and May to July 2013 (non-breeding season)[19]. Thus, for each period, we had 90 days of interaction data for each collared koala.

During the breeding season, four collared koalas (two male and two female) moved away from the site and recorded no contacts with other collared koalas: these individuals were not included in analyses. This resulted in a dataset consisting of seven males and ten females for the breeding season. During the non-breeding season, 4 collars failed (two male, two female: the proximity units suffered power failures), recording no data, resulting in an analyzable dataset of 14 individuals (four male, ten female) of which one female recorded no interactions with other collared koalas.

We tested for seasonal differences in encounter duration and contact frequency using a two sample t-test with the Analystsoft software [34] and for concordance between morphology and behaviour using Kendall’s Tau [35].

Home ranges were estimated for each individual in the breeding and non-breeding season. We calculated the area of the 93% kernel using a fixed kernel estimator using the LSCV (least squares cross-validation) method to select the optimum smoothing parameter. The 93% fixed kernel was used because the smoothing parameter could not be calculated for all individuals using the 95% kernel. Home ranges were calculated using the package adehabitatHR [36] in the statistical software R [37]. We used two-tailed statistical tests in all cases and defined statistically significant as those cases when P < 0.05.

### Ethics statement

This research was conducted with approval from The University of Queensland Animal Ethics Committee, Animal Ethics Permit number CMLR/304/13/QLD GOVT and San Diego Zoo IACUC permit number 11–029 and was carried out according to the conditions of Queensland Department of Environment and Heritage Protection, Scientific Purposes Permit number WITK12066312.

## Results

**Fig 1:**
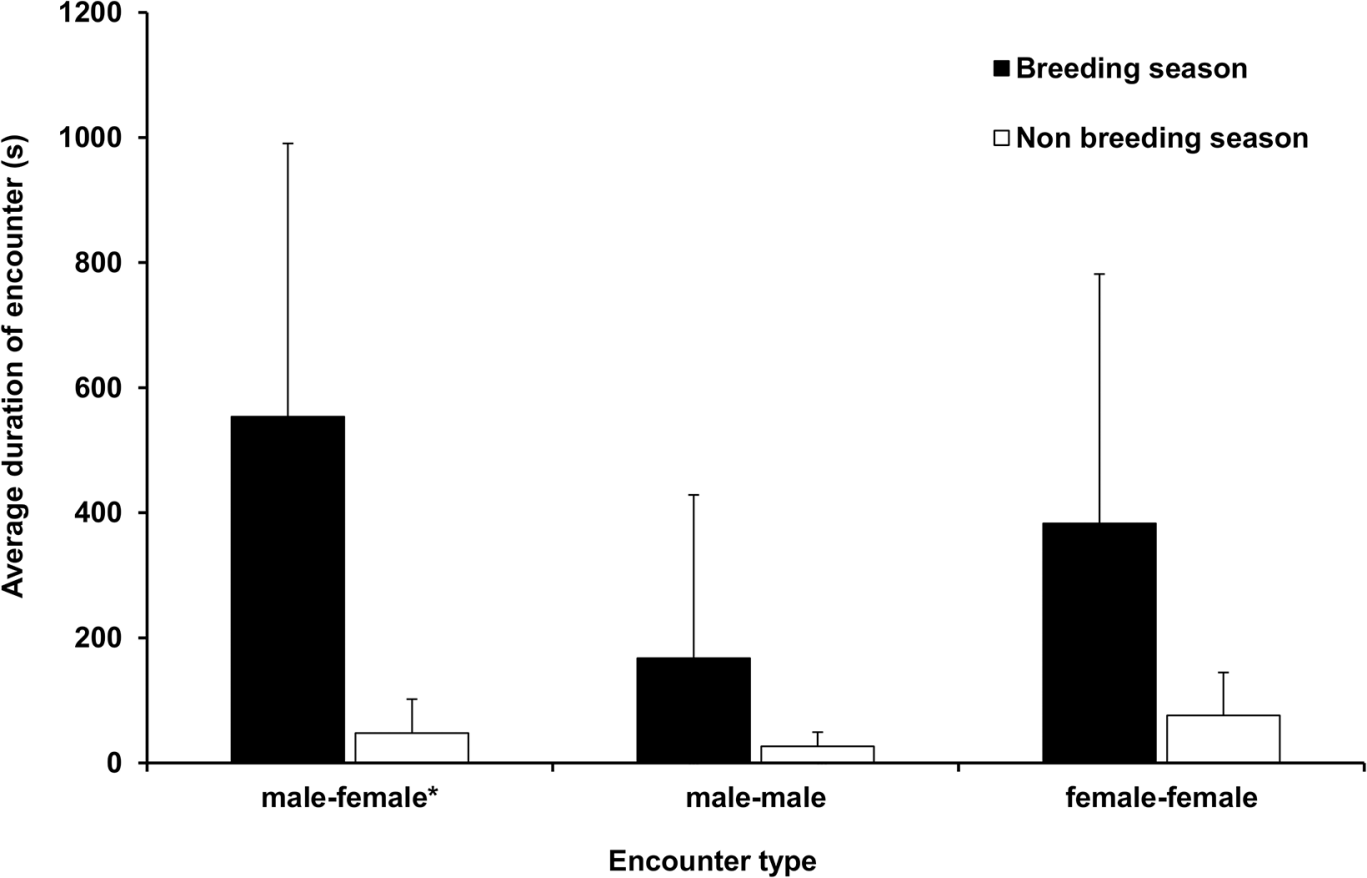
Average encounter duration (± SD) in seconds for koalas at St Bees Island, Queensland, Australia, during breeding (September–December) and non-breeding (May–July) seasons. *P = 0.007

**Fig 2:**
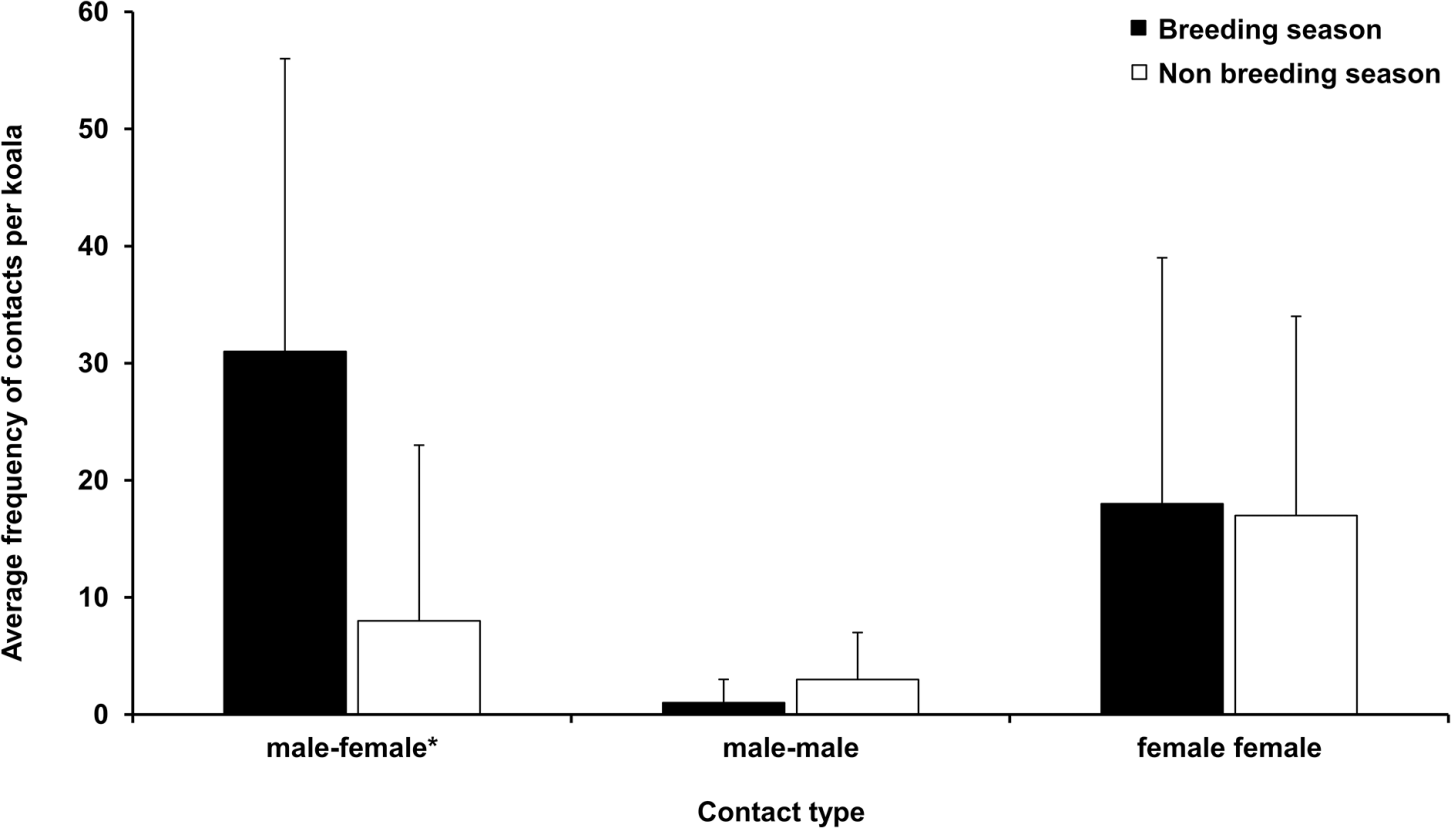
Average contact frequency (mean number of contacts per individual ± SD) for koalas at St Bees Island, Queensland, Australia during breeding and non-breeding seasons. *P = 0.004

We recorded a total of 611 contacts among 224.5 h of reported encounters between 17 koalas (7 male, 10 female) in 90 days of data collection between September and December (the breeding season), compared with 266 contacts among 23.3 h of encounters for the 14 koalas during the same measurement interval starting in May (non- breeding season, Figs 1 and 2).

In the breeding season, 67% (410) of all individual contacts were male-female, 30% (182) were female-female and 3% (19) were contacts between males. In contrast, during the non-breeding months, 39% (105) of contacts were between males and females, with 56% (149) female–female and 5% (12) recorded between males.

During the breeding season, male-female encounters accounted for 66% of total encounter duration, with female-female and male–male encounters accounting for 26% and 8% respectively. By comparison, male-female encounters accounted for 51.5% of encounter duration in the non-breeding season, whereas female-female encounters made up 42% and male-male encounter duration accounted for 6% of total encounter duration.

We had ten female koalas in our study group each season; hence there were 45 different combinations possible. In the breeding season, we observed 11 of the possible female–female combinations. In the non-breeding season we observed ten female–female combinations.

In the breeding season there were 7 males and hence 21 possible male-male combinations, of which 2 were recorded. By comparison, of the 6 possible male–male combinations available (with only four males) in the non-breeding season, only one pair of males interacted.

There were 70 possible male-female combinations during the breeding season, of which 13 were recorded, compared with 6 (of possible 40) combinations recorded during the non-breeding season.

Male variability in the number of possible mates was greater than females during the breeding season: males encountered on average 2.7 females during our three-month analysis of breeding season interactions, compared with females that encountered on average 2 males. The variance in number of females encountered by males was 3.6, compared with 1.25 for females; hence male variance in mate encounters was 2.8 times that for females.

To test for the affect of season on the frequency of male–female contacts, we used a two-tailed t-test where individual koalas that were not represented in both seasons were replaced, when absent, with data from a koala of the same sex and of similar body mass. We did this because age and sex are likely to influence social activity of koalas, so we attempted to have comparable groups of koalas of similar age, sex and size in each season. Hence, we opted for a more stringent and accurate analysis even though such an approach reduced sample sizes. Where no suitable replacement was found, the koalas were excluded, resulting in 14 samples in each season. We found a significant difference between seasons in average contact frequency (average breeding = 31 contacts, average non-breeding = 8 contacts, t _13_ = 2.05, p = 0.004) indicating that there were more contacts between male and female koalas during the breeding season than the non-breeding season. Conducting the same analysis to compare the duration of encounters revealed that during the breeding season (average duration of encounter 22 m 24 s) encounters were of longer duration than during the non-breeding season (average duration of encounter 5 m 45 s; t _13_ = 2.05, p = 0.007).

We used Kendalls Tau to investigate the relationship between male body mass and encounter duration. For both “total” encounter duration (including male–male and male–female) and for male-female encounter duration considered separately, there was no significant correlation between body mass and encounter duration during the breeding season (total encounter duration: Kt = 0.43, p = 0.22, male–female encounters only: Kt = 0.14, p = 0.76).

Home range areas for males were variable during breeding season (min 0.76 Ha, max 68 Ha), and the low number of collars (and hence GPS units) recovered from males (breeding season n = 5, non-breeding season n = 3), compounded our uncertainty, so an accurate description of male ranges was not possible. Female ranges were not significantly different in size between breeding season (n = 9, 1.54 ± 0.21 Ha) and non-breeding season (n = 9, 1.2 ± 0.28 Ha; t_16_ = 1.74588, p = 0.08).

## Discussion

The absence of a seasonal effect on female-female associations, and the presence of a significant seasonal effect on male-female proximity, provides evidence that our proximity logging devices were accurately reflecting natural variation in koala movements throughout the year. Our data indicate that direct male-male competition is probably not occurring in the absence of observations by scientists, because the frequency of male-male associations was low, and constant across seasons. Thus, we provide the first field data to confirm the prediction of Sharpe [23], that males will have higher levels of avoidance than females. In addition, male body mass was not associated with differences in proximity to females, which would have been expected if female koalas responded to size cues in male bellows. Neither male nor female body mass was correlated with range size, which did not vary as a function of season in either sex [27].

Our results are limited to non-observational interactions detected by GPS collars indicating the rates and duration with which two koalas were within one meter of each other. The use of remote signaling devices can provide insights into the sociality of these nocturnal, solitary marsupials, but not the details about what occurred during their proximity. The next step is to unravel the detail of the encounters and determine the biological significance of interaction duration–both for same and inter-sex interactions. Only with intensive surveillance or paternity analysis will we be able to determine whether those interactions that last longer are biologically more significant (e.g. they indicate copulation or even conception or cause mortality or range exclusion for males). Our results strongly suggest that a probable mode of sexual selection in koalas is female mate choice, and a secondary mode could involve female-female competition, given that encounter rates between females increased in the absence of changes in home range area. Female movement distance and male bellow frequency are correlated [4], but we found no evidence that female-female interactions were a consequence of dual attraction to the same male at the same time.

We predicted that if male competition was mediated by agonistic interactions, then male-male encounter rates would be higher during the breeding than the non-breeding season (prediction 1). Our results showed no seasonal change in the frequency of male-male contact (Fig 1) although there was a tendency for breeding season encounters to be of longer duration than those in the non-breeding season (Fig 2). The role of vocalizations and scent marking have long been postulated as drivers of social organization in koalas [38] and our data confirm that physical encounters are infrequent components of competition in this species. That breeding season encounters amongst females were also of longer duration than non-breeding season encounters could indicate intrasexual competition amongst females, perhaps focused on resources that are required for access to mates [39], particularly if there is a link between resources and mate quality (for example, if higher quality males occupy habitat of higher quality and females can monopolise access to these mates). Alternatively, if habitat quality was high, then females might be spending more time near each other while feeding, but only future research on female-female behavior will enable scientists to examine why females spent more time near other females during the breeding season. Investigations of female competition and sexual conflict are absent in the literature of koala behavior, yet females maintain range fidelity, with range overlap among related females that appears to be greater than between unrelated females [10]. Although this behavior could be the expression of a genetic correlation with territorial males [40], we found no evidence that males maintained the same home ranges across years [28].

The operational sex ratio (OSR) at St Bees is unbiased [10], yet we found and collared more adult females than males in both seasons of our study. If the OSR determines which sex competes for mating opportunities [41], one might expect female koalas to compete for access to males. Female competition is present where males provide no parental care [42], as occurs in koalas and in a variety of other species [43,44].

Our data suggest that the difference between variance in male-female encounters for male and female koalas (prediction 2) reflects the variance in progeny for this species previously reported by Ellis and Bercovitch [10] and might potentially reflect the strength of sexual selection for koalas (I_m_) [13]. However, we do not have paternity data for the period of our study and the skew in our operational sex ratio (we collared more females than males in each season) undermine a complete treatment of this question.

Our results support earlier hypotheses predicting that males are able to accurately broadcast honest signals about their size [45] and individual identity [46] to other koalas, thereby avoiding physical conflict with other males, while also attracting females. We found no relationship between male body mass and the likelihood of male—female encounter (prediction 3), a finding that supports previous conclusions that male body mass accounted for only a proportion of male reproductive success in the wild [10]. This result suggests that advertisement of size may be more important to male–male interactions (mediating avoidance) than to sexual advertisement for mates. Since koala vocalizations are also transmissions of identity [46] it is plausible that males pay attention to size of competitors, while females are also attending to cues of uniqueness. This conclusion is further supported by previous studies that show that koala females tend to produce offspring sired by different males across years [10,17]. Different male sires for successive joeys could be an outcome of female mate choice.

Our analysis of seasonal variation in female range size (prediction 4) produced smaller estimated ranges than previously calculated for St Bees Island [28], but our finding of no significant difference between breeding season and non-breeding season range size concurred with that earlier report. There are reports showing that female koalas are more active when bellows are more common [4], and that oestrus females are likely to roam in search of mates [22]. Our GPS loggers recorded location only once per night, hence excursions of short duration may have been undetected and our recording period of 60 days for location data does not encompass the complete breeding or non-breeding season.

Finally, we found that, as predicted (prediction 5), there were more interactions between koalas during the breeding season compared with the non-breeding season, but in contrast to the second tenet of this prediction, female-female encounter duration (but not frequency) differed between seasons. Evidence of seasonal variation in encounter duration for females could indicate a role for this behavior in breeding dynamics, possibly through competition for resources that might attract quality males. However, an understanding of this behavior requires comprehensive knowledge of the age structure and relatedness of individuals in the population; females may simply be more tolerant of other females. Female koalas encounter few males and produce only a single offspring each season, so there may be competition amongst females for access to males, or for residence in habitat of higher quality, if males seek these habitats as well.

Because female koalas generally rear a single offspring annually, male reproductive output should be directly related to the number of mates they pair with over the course of a breeding season [47]. Hence access by males to females could reflect sexual selection in this species. Our remote recording of koala movement and proximity to conspecifics supports earlier findings that koala females probably exercise mate choice by moving to males that are bellowing [4]. Male size information is transmitted in bellows, but body mass can only account for a portion of the variation in siring success. We suggest that sexual conflict, for example, could also influence reproductive success in koalas. Koalas are induced ovulators [48] and Bercovitch [49] has proposed that mechanisms might have evolved that enable females to exercise mate choice through this response. In addition, although females are smaller than males, they do scream and physically defend themselves (scratching and biting) when some males attempt to copulate with them [50] which presumably (though untested for wild koalas) could deter unwanted males or reduce the success of attempts at copulation by such males. We found no evidence of multiple contacts (between any one female and several males in rapid succession), which would indicate a role for sperm competition in koala mating systems, but cannot discount that this is also possible.

We conclude that sexual selection in koalas is complex and that the lower levels of mutual avoidance observed for females compared with males points to female competition for mates in this species. Females are traveling during the breeding season more than males, possibly seeking specific mates. Males appear to use their bellows to attract females as well as to repel other males, since male-male encounter rates do not vary between seasons. Male body mass influences reproductive success, but other factors also regulate fecundity. For example, vocalisations probably travel different distances relative to scents, so the two factors may influence interactions over different distances or in different ways. To date, the influence of koala male scent secretions on reproduction has not been examined, and this represents yet another aspect of koala dimorphism worth investigating, particularly if it also has a role in mediating female choice.

## Supporting Information

S1 File (XLSX)
